# Machine Learning for Precision Health Economics and Outcomes Research (P-HEOR): Conceptual Review of Applications and Next Steps

**DOI:** 10.36469/jheor.2020.12698

**Published:** 2020-05-12

**Authors:** Yixi Chen, Viktor V. Chirikov, Xiaocong L. Marston, Jingang Yang, Haibo Qiu, Jianfeng Xie, Ning Sun, Chengming Gu, Peng Dong, Xin Gao

**Affiliations:** 1 Pfizer Investment Co. Ltd., Beijing, China; 2 Real World Evidence, Pharmerit International, Bethesda, Maryland, United States; 3 Pharmerit (Shanghai) Company Limited, Shanghai, China; 4 Fuwai Hospital, Beijing, China; 5 Zhongda Hospital, Southeast University, Nanjing, China; 6 Easy Visible Sky Tree Technology (Beijing) Co., Ltd., Beijing, China; 7 Sanofi (China) Investment Co. Ltd., Beijing, China

**Keywords:** net monetary benefit, cost-effectiveness, patient heterogeneity, random forest

## Abstract

Precision health economics and outcomes research (P-HEOR) integrates economic and clinical value assessment by explicitly discovering distinct clinical and health care utilization phenotypes among patients. Through a conceptualized example, the objective of this review is to highlight the capabilities and limitations of machine learning (ML) applications to P-HEOR and to contextualize the potential opportunities and challenges for the wide adoption of ML for health economics. We outline a P-HEOR conceptual framework extending the ML methodology to comparatively assess the economic value of treatment regimens. Latest methodology developments on bias and confounding control in ML applications to precision medicine are also summarized.

## INTRODUCTION

Contemporary medical big data open the door for *precision medicine* (also known as stratified medicine and personalized medicine)—that is, evaluating and aligning health care for individual patients based on their disease susceptibility, prognostic and diagnostic information, and treatment response.^1^,^2^ Technological advancements in the availability of big data can play an important role in health economics and outcomes research (HEOR) as well,^3^ where precision medicine applications can help discover and align treatment pathways with the highest likelihood of treatment success and quality of life for specific patient clusters.^1^ Use of the term *precision HEOR* (P-HEOR) has been suggested to broaden the scope of precision medicine. P-HEOR integrates economic and clinical value assessment by explicitly discovering distinct clinical and health care utilization phenotypes to optimize the cost-effectiveness of the use of health care interventions.^4^,^5^

Yet, practical and methodological challenges exist in using medical big data for economic evaluations of precision medicine.^6^,^7^ Challenges relate to the presence of bias and confounding in observational studies, handling of missing data and clinical miscoding, absence of available health state utility values (used to calculate quality-adjusted life years [QALYs]) for population subgroups of interest,^6^ as well as lack of clear evidence on willingness-to-pay thresholds and reimbursement.^7^ While these challenges are also present in traditional applications of health technology assessment, which perhaps may explain why patient heterogeneity is rarely looked into in cost-effectiveness assessments,^8^ making causal inferences in the context of multiple identified P-HEOR cohorts could compound the problem.

Highlighting the aforementioned issues as possible reasons, a recent review identified no published economic evaluations using big data to inform precision medicine.^6^ Literature reviews have also brought up the point that the application of HEOR to precision medicine is still in its infancy.^6^,^7^ This does not mean, however, that development work is not already being conducted to identify opportunities and foundational frameworks for the application of health economics tools to precision medicine, as suggested by Veenstra et al. in Table 1.^7^

As the economic evaluation of observational data and generation of real-world evidence will remain an important topic in precision medicine,^7^ expert opinion suggests that contemporary advanced predictive algorithms such as machine learning (ML) should be explored as advanced tools for decision-making and cost-effectiveness analysis,^6^ consistent with those particular domains outlined in Table 1. For example, when paired with clinical opinion from a health care professional, ML results can produce valuable insights into accelerating clinical workflow and optimizing therapeutic interventions and resource allocation.^9^

Aligned with recommendations to document challenges and potential solutions to support future developments in the application of health economics to precision medicine,^6^ the objective of this article is to highlight the capabilities and limitations of ML applications in P-HEOR through a conceptualized example. We contextualize how a P-HEOR framework centered on recursive partitioning ML methods can be used to examine heterogeneity of treatment effect and to assess patient subgroups in which a particular intervention can be cost-effective versus another. We also summarize the latest methodology developments on how bias and confounding can be controlled for when ML techniques are applied to nonrandomized data for P-HEOR research purposes. While multiple types of ML models are available, this article focuses on the potential P-HEOR implementation of decision tree and random forest (RF) models, which are among the most widely used ML techniques. The clinical context of our conceptualized example is to identify specific P-HEOR subgroups among septic patients in an intensive care unit (ICU) who are at higher risk of longer length of stay (LOS).^10^

The reason why we have chosen sepsis as our example is that it is the leading cause of mortality worldwide^11^ and requires extended stay in an ICU, which can pose significant economic burden.^12^ Within a health care organization, predicting ICU LOS can inform planning to fulfill demand for ICU care (eg, number of beds, staffing).^13^ Regarding the importance of ML techniques in sepsis, a quality improvement team has already successfully implemented an ML-based prediction algorithm during regular care to identify patients with sepsis earlier, with 50% lower readmission rate and 60% lower in-hospital mortality, compared to the pre-implementation period.^14^ In another sepsis study, in-hospital mortality and LOS were significantly reduced in an arm of a randomized clinical trial evaluating the use of an ML-based sepsis surveillance system versus the control sepsis detector.^15^

## DESCRIPTION OF RECURSIVE PARTITIONING METHODS FOR P-HEOR

The benefit of using ML over traditional techniques in precision medicine is that ML determines data-driven nonlinear and nonmonotone association rules by simultaneously processing a large number of predictors. The ML models do not need to be specified in advance, and the combinations of the resulting predictors could generate new evidence for patient subgroups.^16^ One such implementation of ML is RFs.

In short, the RF algorithm is comprised of multiple individual decision trees, where each decision tree represents a statistical model that is applied to a random sample of the analytic dataset. Unlike traditional linear regression models, where results are shown as a linear combination of estimated coefficients in front of the prespecified variables included in the model, results of decision tree models are represented hierarchically as the dataset is sequentially split in a binary fashion and patient cohorts of smaller and smaller sample sizes are identified. The RF methodology also has the advantage of being insensitive to multicollinearity. This is especially true when there are many related candidate predictors, such as multiple composite score indices. For comparison, conventional statistical models assume that the independent variables are not correlated. Such assumptions are often violated as the number of independent variables increases.

The implementation of the tree-growing procedure is controlled, in part, by splitting and stopping criteria such as the number of patient predictors to be selected at random during the tree-growing process. A single decision tree is sensitive to changes in the underlying data, as samples are drawn at random and may result in unstable tree structure and predictions. Practical guidance exists on how to optimize process parameters and criteria.^17^ The RF model is constructed by averaging the predictions across all grown trees to achieve more reliable predictions than single trees, but the forest is difficult to visually interpret. As a balance between interpretability and predictive accuracy, one (or a few) representative individual tree(s) with the smallest dissimilarity in predictions between the tree(s) and the overall forest can be identified and visualized.^18^,^19^ Metrics such as average squared errors in the validation set, out-of-bag estimates of error rates obtained from neighborhoods of representative trees, as well as sensitivity, specificity, and accuracy, can be compared to assess model performance.^19^

**Figure 1:**
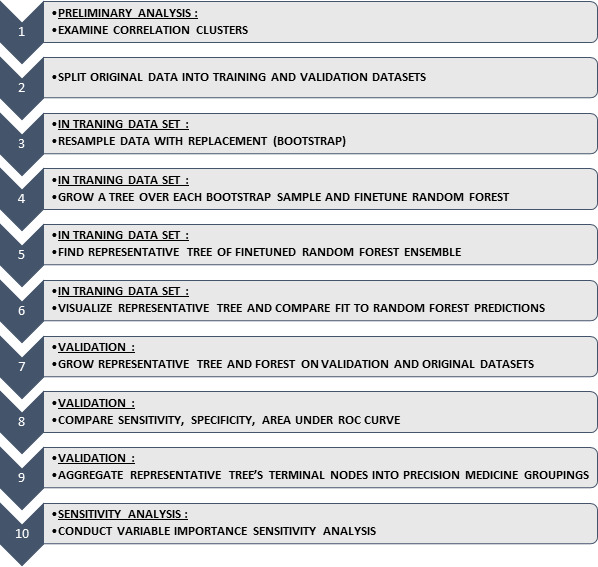
Tree-based algorithm framework that could be adapted to P-HEOR (adapted from Chirikov et al 2017) Adapted from: Chirikov VV, Shaya FT, Onukwugha E, Mullins CD, Dosreis S, Howell CD. Tree-based Claims Algorithm for Measuring Pretreatment Quality of Care in Medicare Disabled Hepatitis C Patients. *Med Care*. 2017;55(12):e104–e112.

Previously, we outlined an easy-to-follow 10-step framework (Figure 1) on the use of RF models in health services research,^18^ which visualizes results in a decision tree format that is naturally aligned to assist clinical interpretation by physicians engaging patients in the decision-making process.^18^,^20^

The intent behind the framework is to do an exploratory correlation analysis of patient characteristics of interest, split the data in separate training and validation datasets, then grow and tune the parameters of the RF in the training dataset, and determine and visualize the representative tree of the RF that results in the predictions most similar to the overall forest. Only after comparing the predictive accuracy in a validation dataset can the representative tree’s terminal nodes be aggregated into logical precision medicine groupings. The last step is sensitivity analysis on the relative importance of each of the patient characteristics in the RF model. Detailed descriptions of the steps of the algorithm are presented in Table S1. For a comprehensive application of the algorithm, we encourage readers to refer to previously published work.^18^

We first show an example of how this RF model framework can be applied to identify patients with sepsis at high risk of longer LOS. The example covers stratification of the patient sample, as driven by the RF partitioning rules, into subcohorts of patients with higher and lower probability of LOS. We do not compare treatment agents for sepsis, do not attempt to make causal inferences, and do not conduct a formal cost-effectiveness analysis. We do, however, use our example as the technical foundation to introduce recent methodological developments and conceptually outline how the approach can be leveraged for P-HEOR in the near future.

## EXAMPLE OF RANDOM FORESTS APPLICATION

We used data from the large, publicly available Medical Information Mart for Intensive Care (MIMIC) III database comprising deidentified health-related data associated with over 40 000 patients who stayed in ICUs of the Beth Israel Deaconess Medical Center (Boston, MA) from 2001 through 2012. The database includes information on demographics, vital signs, laboratory test results, imaging reports, procedures, medications, and outcomes.^21^ For data management and creating the analytic dataset, we used SAS 9.4 (Cary, NC). For implementation of the RF, we used the caret (Classification And Regression Training) and party (A Laboratory for Recursive Partytioning) packages in R software (R Foundation for Statistical Computing, Vienna, Austria).

The study cohort included sepsis survivors aged 18 years or older with complete data for all study variables. A total of 1474 patients were eligible for the study (Figure S1). The endpoint was patients’ risks of having a prolonged stay in ICU, defined for practical purposes as a LOS ≥6 days, which was the observed median LOS in the sample. There were 656 (44.5%) septic patients who stayed at least 6 days in ICU.

Compared to sepsis patients without extended ICU stays, patients with extended ICU stays appeared to be younger (60.85 vs 64.64 years of age), more were privately insured (35.8% vs 26.3%), and more had surgical ICU admissions (surgical ICU 17.5% vs 12.8%; trauma/surgical ICU 14.6% vs 7.0%; surgery recovery unit 5.8% vs 2.8%). Patients with extended ICU stay had higher baseline rates of obesity (11.3% vs 5.1%) and cardiac arrhythmia (38.9% vs 32.4%), but lower rates of metastatic cancer (7.0% vs 13.1%). Patients with extended ICU stay scored higher on shock index (1.37 vs 1.17), systemic inflammatory response syndrome (SIRS) criteria (3.30 vs 3.20), simplified acute physiology score (SAPS) II (46.0 vs 41.4), acute physiology score (APS) III (60.0 vs 54.1), logistic organ dysfunction score (LODS) (6.68 vs 5.25), sequential organ failure assessment (SOFA) (7.28 vs 5.83), and Oxford Acute Severity of Illness Score (OASIS) (40.7 vs 35.5).

Patient characteristics included in the algorithm-building process were demographics, laboratory values, and clinical characteristics captured from 12 hours before to 24 hours after the ICU admission. The RF was grown and the representative tree was extracted as per the 10-step framework previously described.^18^ In terms of the general accuracy, the overall RF model outperformed the representative tree model with an accuracy level of 0.7719 versus 0.7249 in the training set and 0.7273 versus 0.6955 in the testing set (Table 2). While the RF model resulted in more reliable predictions than its representative tree, the RF was difficult to interpret as individual trees are lost in the overall forest.

**Figure 2:**
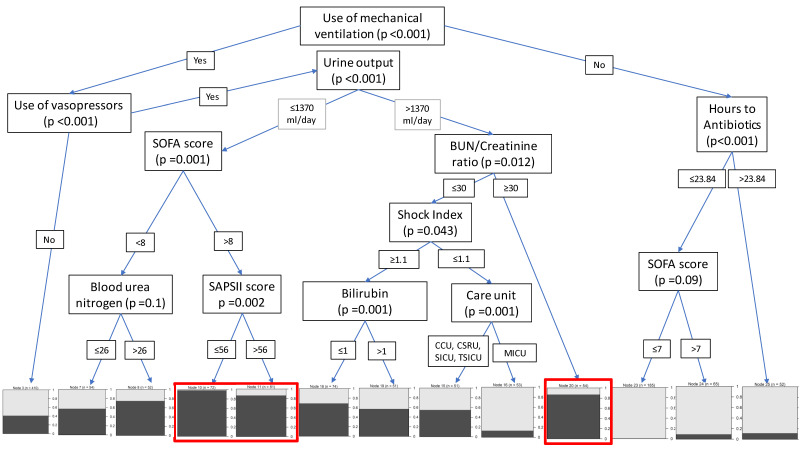
A Representative Tree of the Random Forest (RF) Model^a^ ^a^Shaded boxes represent the probability of a prolonged stay in ICU, defined as the median LOS ≥ 6 days. The red boxes highlight the patient groups with the highest probability of a prolonged LOS in ICU. Abbreviations: BUN, Blood urea nitrogen; SOFA, Sequential Organ Failure Assessment; SAPSII, Simplified acute physiology score (SAPS) II. Care units: MICU, Medical Intensive Care Unit; SICU, Surgical Intensive Care Unit; CCU, Cardiac Care Unit; CSRU, Cardiac Surgery Recovery Unit; TSICU, Trauma Surgical Intensive Care Unit.

Figure 2 illustrates the representative tree of the RF model. As shown in the tree, patients were categorized into different risk groups based on selected features. High-risk patients were defined as having greater than 80% probability of having prolonged ICU stay. In the representative tree, two groups of patients had the highest risk of staying more days in ICU: the first group of high-risk patients used mechanical ventilation, used vasopressors, had urine output ≤ 1370 mL/day, and had a SOFA score >8; the second group of high-risk patients also used mechanical ventilation and vasopressors, had urine output >1370 mL/day, and had a BUN/creatinine ratio >30. Of note, SAPS II at a threshold >56 or <56 produced two statistically significantly different subcohorts among the first group of patients, but we kept those subcohorts together as we deemed that they did not result in clinically significant differences in the probability of having longer LOS. Other gradients of increased probability of longer LOS were also indicated and were associated with SOFA score <8 or BUN/creatinine ratio <30, among those with mechanical ventilation. No use of mechanical ventilation had the lowest probability of extended LOS.

Figure S2 shows the importance level of each predictor on extended stay from the sensitivity analysis. Using mechanical ventilation was the most important prognostic feature, followed by composite severity scores (eg, LODS, APS III, OASIS, and SAPS II). Vasopressor use was also among the top prognostic features. Other identified clinical features were consistent with the literature.^14^,^22^

## CONCEPTUAL FRAMEWORK FOR IDENTIFYING P-HEOR COHORTS

In the application of the RF methodology described previously, the representative tree was easy to interpret, as high-risk patients were visualized into separate groups based on their characteristics. We would like to introduce the concept that similar RF methodology can be applied to modeling all-inclusive HEOR outcomes of interest, such as incremental cost-effectiveness ratios (ICER) and net monetary benefit (NMB).

Less than 20 years ago, Hoch, Willan, and Briggs introduced the net benefit regression framework that combined the statistical approaches of health econometrics with cost-effectiveness analysis (CEA)^23^ and allowed researchers to tackle HEOR research questions using observational data by allowing them to monetize treatment benefit as well as adjust for imperfect randomization or other covariate imbalances. In general terms, for two treatments it can be expressed as

$$\Delta \text{NMB}={\text{NMB}}_{1}-{\text{NMB}}_{0}=\lambda \cdot ({\text{E}}_{1}-{\text{E}}_{0})-({\text{C}}_{1}-{\text{C}}_{0})$$

where subscript 1 designates the treatment of interest, subscript 0 designates the standard of therapy arm, λ is the willingness-to-pay parameter of the decision maker, the difference (E_1_–E_0_) is the average difference in clinical outcomes, and the difference (C_1_–C_0_) is the difference in average total costs. The incremental NMB (ΔNMB) is positive when the monetary valuation of treatment benefits outweighs the incremental costs, as well as when the cost savings of using a cheaper standard of therapy are greater than the decrement in forgone clinical benefits (eg, due to an innovative experimental therapy). A follow-up article introduced an extension to the net benefit regression framework by allowing for adjustment for prognostic variables and examination of prespecified subgroups with potentially differential ΔNMB.^24^

**Figure 3:**
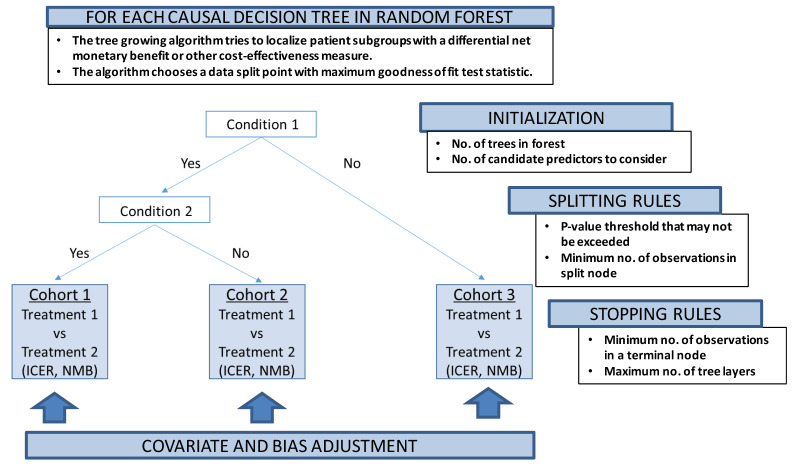
Conceptual Illustration of Causal Decision Tree to Identify P-HEOR Subgroups with Differential Cost-Effectiveness Outcomes Abbreviations: No., Number; ICER, Incremental Cost-Effectiveness Ratio; NMB, Net Monetary Benefit.

Our proposed conceptual P-HEOR framework is a data-driven approach using model-based extension of the RF methodology as the technical foundation to reveal clinically relevant patient groups in which both differential treatment effects and costs are optimized (Figure 3).^25^ This idea follows up on other recent work supporting innovation in regression-based approaches to patient-centered CEA,^26^ as well as subgroup and individual treatment effect prediction using recursive partitioning RF.^27^,^28^

Model-based recursive partitioning RF produces a segmented model in which patient subgroups emerge with differential treatment effects,^28^,^29^ if treatment regimens are also included in the set of predictors. The implementation of the tree-growing procedure is again controlled by multiple parameters, similar to the algorithm described in the previous section, which can be fine-tuned by iteratively varying the values of each parameter and empirically selecting the combination of parameters yielding the lowest prediction error.^17^

The treatment effect, say, between intervention A and B could be covariate- and bias-adjusted for potential imbalance between underlying patient characteristics using randomized clinical data,^30^ as well as if applied to nonrandomized observational data.^31^ Future developments in the implementation of ML algorithms will not only allow for covariate adjustment,^32^ but also control for informative censoring of costs via propensity score weights^33^ and provide an overall flexible statistical framework able to handle adjusted subgroup identification among two or more treatments.^34^ For example, Yang et al. have recently applied their causal interaction trees methodology, which incorporates inverse probability weighting, g-formula, and doubly robust estimators during the decision tree construction, to the observational Study to Understand Prognoses and Preferences for Outcomes and Risks of Treatments (SUPPORT).^35^ The authors found that the treatment effect of right heart catheterization depended on the 2-month probability of surviving, similar to other published research that examined this criterion in prespecified subgroup analysis.^36^

## DISCUSSION

While most applications of HEOR and CEA to precision medicine have focused on pharmacogenomics^37^ to assess the value of precision testing to diagnose patients and align them with the appropriate targeted oncologic agent(s),^7^ there is still a paucity of published research evaluating which precision patient subgroups can benefit from a particular cost-effective treatment regimen.^6^ Some of the reasons are methodological and relate to our core understandings of bias and confounding. Individual treatment effect prediction could be hindered when predictive patient characteristics are also influencing treatment assignment^27^ or when the scales for capturing a particular covariate are subject to measurement error, such as in mental health conditions.^38^ In clinical drug development for precision medicine, while most novel analytic methods may perform well in cohorts with large sample sizes and sizable treatment effects,^39^ in real-world scenarios many of these methods have been found to perform poorly (as perhaps traditional statistical approaches would) on at least one of seven criteria: (i) bias in selection of subgroup variables, (ii) probability of false discovery, (iii) probability of identifying correct predictive variables, (iv) bias in estimates of subgroup treatment effect, (v) expected subgroup size, (vi) expected true treatment effect of subgroups, and (vii) subgroup stability.^40^

In our own example of applying the RF methodology to a cohort of patients with sepsis, we likely encounter the same issues we have mentioned. Additionally, by focusing on survivors only, our example is not representative of the general population of sepsis patients in ICU. However, we do hope that with future methodological developments, such as the application of causal interaction trees in the RF or other ML ensemble methods,^40^ novel methods would be seen as less of a black box and subject to criticism.^6^,^41^ Going forward, achieving unbiased variable selection, unbiased estimates of subgroup treatment effects, and probability of false discovery,^40^ as well as the publication of several case studies on prospective, controlled, and transparent validation,^42^ would be paramount before we see such techniques more frequently used for P-HEOR. This is not to suggest that there are no attempts to generate foundational evidence for the next wave of P-HEOR. A prime example of this is the Personalized Risk Information in Cost-Effectiveness Studies (PRICES) project, funded by the National Institutes of Health.^7^ Using risk models, the project aimed to understand how each patient’s individualized risk of experiencing adverse health outcomes can help clinicians better tailor care and resources.^43^ Additionally, ML analyses seem to be gaining traction as the toolbox of interest to reanalyze randomized clinical trials, where the risk of treatment selection bias may be somewhat limited.^44^,^45^

The expectation is that with the pace of analytic innovation, many of the issues we have raised could be resolved in the years to come. We have presented a P-HEOR conceptual framework that is built on RF ensemble methods but also has the objective of identifying a single representative decision tree in which clinically interpretable subgroups could be used for individualized prediction. The successful application of the P-HEOR conceptual framework would rely on the granular availability of life expectancy, utility, and costs so that they are representative of the identified unique precision subgroups. This is important as input choice solely based on population averages may widely influence value-based decision-making for individual patients.^8^ Last but not least, use of the framework would be dependent on the wider adoption of a broader concept of value including moving toward a “net monetary benefit” comparison by monetizing the QALY as well as agreeing via multicriteria deliberative processes what the most appropriate willingness-to-pay thresholds λ would be for each precision cohort.^46^

## CONCLUSIONS

In the era of big data, P-HEOR can benefit from ML optimization to identify patient cohorts with different risk-benefit profiles in terms of both clinical and economic outcomes. We propose a conceptual P-HEOR framework that holds the promise of precisely assessing the value of a specific treatment in heterogeneous subgroups using realworld evidence. However, methodological challenges still remain before we see a wave of reliably conducted P-HEOR analyses. As prediction accuracy in health economics is always highly dependent on the quality of the data and methods, future efforts should focus on applying the latest statistical and framework innovations for value assessment to high-quality real-world data.

## Supplementary Information



## Figures and Tables

**Table 1:** HEOR Applications to Precision Medicine as per Veenstra et al (2020)

Decision analysis and simulation modeling	Trial-based economic evaluation	Cost-effectiveness analysis
Quantify uncertainty and elucidate therapies’ benefits and harms in the absence of direct evidence	Direct estimates of therapies’ effect on healthcare economic burden and clinical outcomes	Inform decision-making with regard to treatment choices and reimbursement

**Table 2:** Predictive Performance Comparison

Machine Learning model	Random Forest (RF)		Representative Tree from RF^a^	
Parameter	Train Dataset	Test Dataset	Train Dataset	Test Dataset
Accuracy	0.7719	0.7273	0.7249	0.6955
Kappa	0.5384	0.4412	0.4387	0.3729
Sensitivity	0.7455	0.6327	0.6523	0.5714
Specificity	0.7931	0.8033	0.7830	0.7951
Positive Predictive Value	0.7429	0.7209	0.7068	0.6914
Negative Predictive Value	0.7954	0.7313	0.7375	0.6978
Balanced Accuracy	0.7693	0.7180	0.7177	0.6833

NOTE:

a the best-performing tree in the forest
