# Supplementary material for: Machine Learning for Precision Health Economics and Outcomes Research (P-HEOR): Conceptual Review of Applications and Next Steps
Source: J Health Econ Outcomes Res. 2020 May 12;7(1):35–42. doi: 10.36469/jheor.2020.12698 (PMC7299485; doi:10.36469/jheor.2020.12698)
Supplement: Supplementary file 1 [file jheor-7-2-12698-s01.pdf]

JHEOR Journal of Health Economics and Outcomes Research

## Supplementary Online Material

Chen Y, Chirikov VV, Marston XL, Yang J, Qiu H, Xie J, et al. Machine learning for precision health economics and outcomes research (P-HEOR): conceptual review of applications and next steps. *JHEOR*. 2020;7(1):35-42. doi: 10.36469/jheor.2020.12698

**Supplemental Figure 1.** Patient flow chart of meeting inclusion/exclusion criteria for sepsis cohort used in the conceptual example.
**Supplemental Figure 2.** Predictor Importance in the Random Forests Model
**Supplemental Table 1.** Comprehensive Description of Random Forests Algorithm Steps.

This supplementary material has been provided by the authors to give readers additional information about their work.

This is an open-access article distributed under the terms of the Creative Commons Attribution 4.0 International License (CCBY-4.0). View this license's legal deed at http://creativecommons.org/licenses/by/4.0 and legal code at http://creativecommons.org/licenses/by/4.0/legalcode for more information.

**Supplemental Figure 1.** Patient flow chart of meeting inclusion/exclusion criteria for sepsis cohort used in the conceptual example.

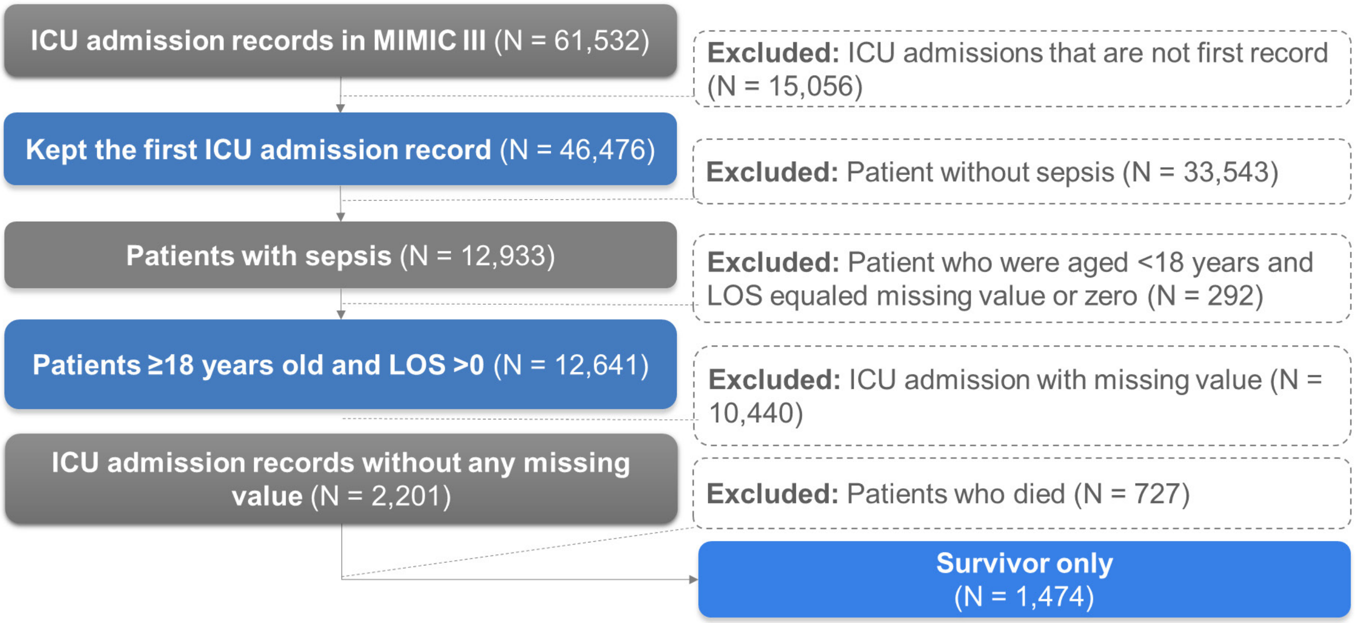


**Supplemental Figure 2.** Predictor Importance in the Random Forests Model

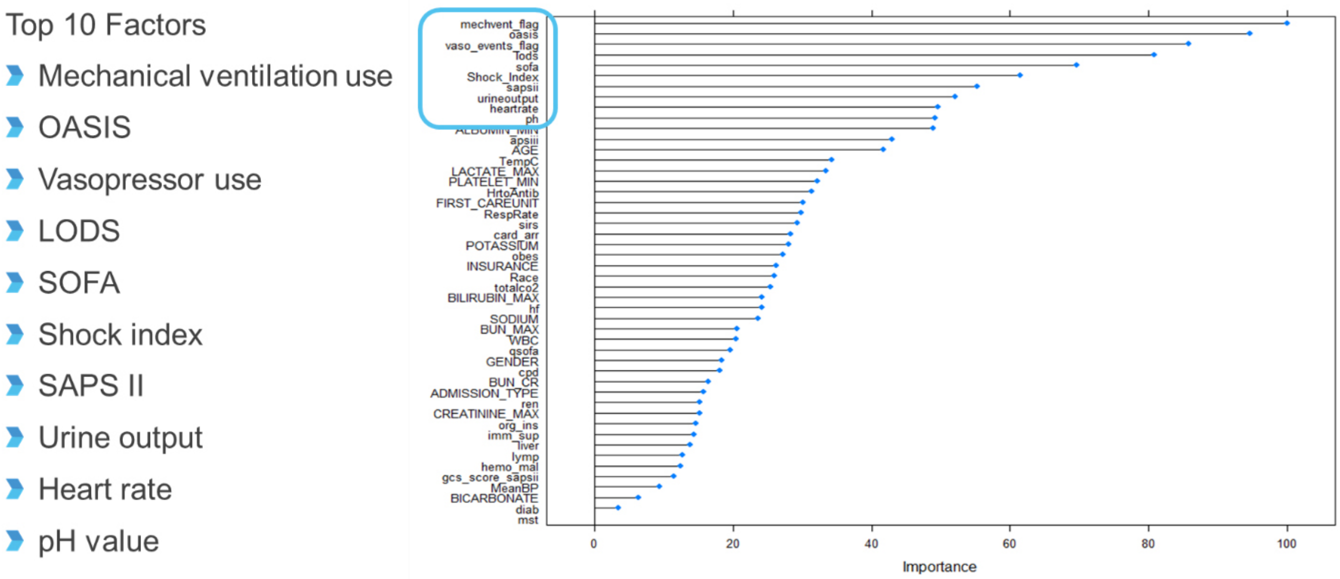


Abbreviation: OASIS= Oxford Acute Severity of Illness Score ; LODS=Logistic Organ Dysfunction Score; SOFA=Sequential Organ Failure Assessment; SAPS II=Simplified Acute Physiology Score II

**Supplemental Table 1.** Comprehensive Description of Random Forests Algorithm Steps.

<table>
<tr><th>ALGORITHM STEP</th><th>RATIONALE</th></tr>
<tr><td>1. Examine correlation clusters among predictors</td><td>In preliminary analysis, use hierarchical cluster analysis to explore the correlations between individual covariates/characteristics of interest. Characteristics can be clustered according to their average similarity distance, calculated as the square root of sum of squares of differences in pairwise correlations. They can be plotted in a hierarchical clustering dendrogram.<br><br>This preliminary step helps generate intuition and expectation as to how next steps of the suggested algorithm breaks data into meaningful groupings and provides a preliminary benchmark against which the final step of the algorithm can be compared against.</td></tr>
<tr><td>2. Split data into a training (learning) and validation data sets via stratified random sampling of the outcome variable</td><td>The original data can be split into training : validation data sets ratio of 75:25 as per a scaling law for the validation-set training-set size ratio and current data mining practice standards.</td></tr>
<tr><td>3. In the training data set, create N versions of data set using resampling with replacement (bootstrap)</td><td>Bootstrapping is an uncertainty modelling step that generates modified versions of the training set over which trees will be grown and ensemble predictions averaged. A size of 1000 bootstrap versions of the data (chosen number of trees to be grown in the forest) can be considered large enough to achieve robust predictions as shown.</td></tr>
<tr><td>4. Grow a tree over each bootstrap sample (random forest) and fine-tune ensemble</td><td>Fine-tuning random forest ensemble parameters:
<table>
<tr><th>PARAMETER</th><th>GUIDANCE</th><th>SUGGESTED DEFAULT</th></tr>
<tr><td>No. of trees</td><td>Choose number of trees large enough to ensure prediction error stabilizes</td><td>N=1000</td></tr>
<tr><td>No. of candidate predictors</td><td>Optimal value depends on the data at hand. Assess predictive accuracy of the ensemble at different values and choose the one yielding the best prediction.</td><td>Vary range from min to max number of predictors in interval of 3 and choose the ensemble that maximized the area under the receiver operating curve (ROC)</td></tr>
<tr><td>P-value threshold to split a node</td><td>Optimal value depends on the data at hand. Assess predictive accuracy of the ensemble at different values and choose the one yielding best prediction</td><td>Run each random forest ensemble at the following p-value thresholds: a) p=0.05, b) p=0.20, c) p=0.40 and choose the ensemble that maximizes the area under the receiver operating curve (ROC)</td></tr>
<tr><td>Minimum size of node to be split</td><td>Recommendation is to set parameter to a small value. A large value may prevent the selection of categorical predictors with large and small categories.</td><td>Minimum size of node to be split can be as little as n=20 or n=40</td></tr>
<tr><td>Minimum size of terminal nodes</td><td>Seen as a tuning parameter controlling size of each tree.</td><td>Minimum size of the terminal nodes could be set at little as n=50 or n=100</td></tr>
<tr><td>Maximum no. of layers</td><td>Not restricted</td><td>Let other chosen parameters control tree size</td></tr>
<tr><td>Resampling scheme</td><td>With or without replacement schemes would lead to similar results for predictors with similar number of categories</td><td>When using modified versions of the training set, resampled estimates of model performance can become biased, as some observations are chosen more than once.<br><br>The proportion of resampling can be set to 63.2%, the theoretical average proportion of observations included in a bootstrap sample drawn with replacement, to control for prediction bias.</td></tr>
</table></td></tr>
<tr><td>5. Find the representative tree of the random forest based on closeness of prediction</td><td>Identify and extract the most representative tree of the random forest, based on the smallest difference in predictions between the tree and the forest (using a metric such as Banerjee et al's mismatch percentage distance metric)</td></tr>
<tr><td>6. Visualize representative tree and compare fit to random forest ensemble</td><td>Before moving onto the next steps of the algorithm, ensure that the representative tree and the random forest achieve comparable predictive accuracies in the training (learning) data sample.</td></tr>
<tr><td>7. Grow representative tree and random forest on validation and original data sets</td><td>Using the validation and the original data sets, obtain predictions by the representative tree and the random forest ensemble.</td></tr>
<tr><td>8. Compare sensitivity, specificity, and area under Receiver Operator Characteristic curve (ROC)</td><td>Examine if the predictive accuracy of the representative tree is comparable to that of the ensemble of trees and is consistent across the original, training, and validation data sets.</td></tr>
<tr><td>9. Aggregate representative tree's terminal nodes based on similar predictions</td><td>The final nodes of the representative tree define a possible precision medicine classification scheme and a combination of final nodes to subgroups might be indicated.</td></tr>
<tr><td>10. Conduct sensitivity variable importance analysis</td><td>The relative variable importance measure in random forests can be obtained. It is defined as the difference in prediction error resulting from random permutation of the values of a predictor variable (averaged over all trees and evaluated together with the remaining unpermuted predictors) and the prediction error before the permutation. The main advantage of the random forest permutation accuracy importance is that it provides a measure of each variable's importance in the context of multivariate interactions with other predictors that might have gone unnoticed in single trees or parametric regression models.</td></tr>
</table>
